# Comparison of Online Dementia Information in Chinese and in English Languages

**DOI:** 10.7759/cureus.1808

**Published:** 2017-10-28

**Authors:** John T Tsiang, Benjamin K Woo

**Affiliations:** 1 Medicine, Case Western Reserve University School of Medicine; 2 UCLA

**Keywords:** chinese americans, asian americans, dementia, google, alzheimer's disease, health information, internet, prevention, chinese language, social media

## Abstract

Introduction

There is a deficit of avenues for obtaining dementia information in the Asian American community. This study aims to compare the content and quality differences between websites providing information on dementia as found by a Google search conducted both in simplified Chinese characters and in English.

Methods

A Google search was performed for the phrase “dementia” in simplified Chinese characters and in English. The resultant websites were categorized by whether they were commercial in nature, the type of website, and whether the website provided an explanation of dementia signs and symptoms. The quality of the websites was assessed via readability and the Health on the Net Code of Conduct (HONcode). Chi-squared analyses were performed to establish differences between the English and simplified Chinese results.

Results

The simplified Chinese search websites were more likely to be commercial (p=0.045) and were more likely to not meet HONcode standards (p=0.008). No statistical significance was observed between the types of websites (p=0.127), the prevalence of signs and symptom explanations (p=0.073), and the readability of the website (p=0.151).

Conclusion

The quality of websites obtained from the simplified Chinese character Google search was lower than those obtained from searches using the English language. Given the limited sources of language and culturally appropriate information on dementia, improvement of Internet resources may help to improve health outcomes of dementia patients in the Asian American population.

## Introduction

As the elderly population in the United States increases, the number of Americans diagnosed with dementia is likewise projected to grow. Data from the Alzheimer’s Association estimates that at least one in ten adults age 65 and older is diagnosed with some form of dementia [1]. With Asian Americans as the fastest growing ethnic group over the last decade, it is becoming increasingly important for individuals in this ethnic community to be informed about this medical condition [2].

Although dementia cannot be completely cured, research shows that behavioral interventions such as cognitive rehabilitation or memory training are most effective in slowing the rate of cognitive decline when implemented in the early stages of the disease. Patients and family members who are more knowledgeable about dementia are more likely to recognize early symptoms and seek professional help during its onset. Unfortunately, studies have also revealed that Asian Americans are significantly less knowledgeable about dementia and are more likely to underutilize its treatment services [3-7]. Information about dementia accepted by this ethnic group often stem from unreliable sources such as personal observations, media, and oral tradition [8-10]. Thus, Asian Americans may be misguided in their beliefs about the symptoms and causes for dementia, which may hinder them from seeking professional help. This finding suggests that proper educational materials and services may be lacking among the Asian American community.

A recent study has found that although Chinese Americans hold misconceptions about dementia, their lack of knowledge does not reflect a lack of desire to become further educated about this medical condition [3]. Moreover, other recent studies have demonstrated that Chinese Americans are becoming more receptive to e-health and to obtaining dementia knowledge using the Internet than before [11-13]. In 2014, Google occupied 69% of the global search engine market and was the most popular search engine in the United States [14]. To the author’s knowledge, there has been no research to date on the content and quality of dementia information available online in Chinese language. Our goal was to compare the content and quality differences between websites providing information on dementia as found by a Google search conducted both in simplified Chinese characters and in English.

## Materials and methods

A list of websites for this investigation was obtained by entering the term “dementia” into www.google.com/ncr for the English search. For the Google search on dementia conducted in simplified Chinese characters, the term “痴呆症” (chīdāizhèng), was entered into www.google.com/ncr, with simplified Chinese as the language used in search settings. The browsing history and cookies were cleared before each search, and the searches were conducted in Google Chrome’s incognito mode to prevent browsers from remembering search activities. Each search was limited to the first three pages of results, as studies indicate that ninety-one percent of internet users do not go past the first page of search results [15]. The Google Chrome web browser was used to navigate each webpage. All videos and downloadable information were included in the evaluation. The information provided on links to external sites was not included in the evaluation.

The content of dementia websites was assessed in the following ways. First, the websites were divided into two categories according to the suffix and their affiliation statement: commercial or non-commercial. Second, each website was categorized into the following types of websites: informational, blog, news, and others (e.g., video, e-commerce, etc.) Third, the websites were reviewed for explaining signs and symptoms of dementia.

The quality of dementia websites was assessed for subjective readability and coherency in the respective search language. The Health On the Net Code of Conduct (HONcode) was also utilized to determine the quality of the websites. The HONcode is a process metric that evaluates medical and health websites in 35 languages with the following eight criteria: authoritative, complementarity, privacy policy, attribution (reference criteria and date), justifiability, transparency, financial disclosure, and advertising policy [16].

All data were entered and analyzed using IBM SPSS Statistics v. 21.0 (IBM, Armonk, NY). The results of the Google search conducted in simplified Chinese characters were compared with the results of the Google English search using chi-squared statistics to assess the content and to measure the quality of dementia information.

## Results

The English google search of the term “dementia” generated 31 hits from the first three pages of search results. In terms of the content of dementia websites, one (3.2%) of the websites was commercial. There were 18 (58.1%) informational, four (12.9%) blogs, four (12.9%) news, and five (16.1%) other types of websites. Twenty-one (67.7%) websites explained signs and symptoms of dementia. For the English search, all 31 (100%) websites were readable in the English language. Twelve (38.7%) of these websites achieved the HONcode quality measures.

For the Google search conducted in simplified Chinese characters, there were also 31 results generated from the first three pages of search results. In terms of the content of dementia websites, six (19.4%) of the websites were commercial. There were 12 (38.7%) informational, five (16.1%) blogs, seven (22.6%) news, and seven (22.6%) others types of websites. Fourteen (45.2%) websites explained signs and symptoms of dementia. For the Chinese search, 29 (93.5%) websites were readable in simplified Chinese characters. Three (9.7%) websites achieved the HONcode quality measures.

In comparison with results from Google’s English search, the websites from Google’s simplified Chinese search were more likely to be commercial (chi-square=4.026, df=1, p=0.045). No statistically significant difference was observed with the types of websites (chi-square=2.325, df=1, p=0.127) nor with whether the website explains the signs and symptoms of dementia (chi-squared=3.215, df=1, p=0.073). While the statistics did not reveal (chi-square=2.067, df=1, p=0.151) overall differences in readability, there were two websites in a different language found using the Chinese search, suggestive of poor search quality. There was a significant difference in the number of websites meeting the HONcode requirements between the two languages (chi-square=7.123, df=1, p=0.008).

## Discussion

This study compared the content and quality differences between websites providing information on dementia as found by a Google search conducted both in simplified Chinese characters and in English. There were significant differences found between the results of English and simplified Chinese Google searches for the term “dementia”. The results in simplified Chinese were significantly more likely to be commercial and were significantly less likely to meet HONcode quality requirements when compared to the results in English.

The significant differences between the number of commercial websites appearing in simplified Chinese and English Google searches suggests that the search engine using simplified Chinese is less able to pinpoint relevant medical knowledge results for the user. This would likely cause more unrelated websites (commercial websites) to appear. For example, two of the six Chinese commercial websites in our search results were links to online marketplaces: one search result was for an anti-aging cream and other beauty products, and the other one was a wrist strap with the word “dementia” in Chinese. Additionally, two of the simplified Chinese Google search results were in a different language (not simplified Chinese). Thus, there is a need for further refinement of the Google search algorithms for simplified Chinese results.

For this study, we used HONcode qualifications as a proxy measurement for website quality. The significantly lower number of websites meeting HONcode qualifications suggests that websites for Chinese readers are less effective in providing appropriate and relevant health information. This lack of quality compounds the fact that Chinese readers, particularly those in the United States, already lack appropriate avenues for obtaining language and culturally appropriate health information on dementia [3,5]. It can thus be inferred that the lack of knowledge regarding dementia may also stem from a lack of adequate Internet sources, alongside many other issues. Future studies should assess stigma and attitudes toward dementia that are portrayed online in Chinese-language websites [17-20].

While there were no significant differences found between the number of websites explaining the signs and symptoms of dementia, only 45.2% of simplified Chinese websites did so, compared to 67.7% of English websites. This suggests that there is some room for improvement in the content of Chinese websites with regard to providing dementia information. As gender and age differences are associated with understanding the prognosis of dementia and identifying the causes of dementia, future studies should incorporate the effects of age and gender on Chinese-language website comprehension [21-22].

Due to the reliance on a search engine to produce the results, limitations of this study revolve around the deficits of the Google search algorithm. It is unknown how well optimized the search engine is for English and simplified Chinese results. The search engine algorithm ranks website relevancy non-randomly. Thus, the sample of websites on dementia obtained in this study is not representative of the entire population of available websites on dementia across the entire Web. The statistics provided should thus not be viewed as an embodiment of all websites on dementia, but rather as a measure of what users of the Internet are likely to see when performing a search on dementia in either language. Further studies should also utilize other search engines to replicate such results. As social media can bridge the gap between health care utilization and the Chinese over cultural barriers, future studies should examine the quality and content regarding dementia on social media [23-24].

## Conclusions

Google search results for "dementia" in simplified Chinese, “痴呆症” (chīdāizhèng), are more likely to be commercial in nature and also of lower quality than Google results for "dementia" in English. Whether this is the result of a lack of optimization in the simplified Chinese search algorithm or a lack of quality websites for obtaining dementia information, the resulting void of information may contribute to the lack of knowledge on dementia and dementia treatment resources in Asian Americans. Our research suggests the need for the following further research: first, future studies should assess stigma and attitudes toward dementia that are portrayed online in Chinese-language websites; second, additional research should incorporate the effects of age and gender on Chinese-language website comprehension; lastly, further studies should examine the quality and content regarding dementia on social media. Ultimately, improvement of web resources on dementia can lead to improvements in knowledge and, therefore, outcomes of dementia in the Asian American community.
